# Electronic Health Literacy Scale-Web3.0 for Older Adults with Noncommunicable Diseases: Validation Study

**DOI:** 10.2196/52457

**Published:** 2024-06-03

**Authors:** Wenfei Cai, Wei Liang, Huaxuan Liu, Rundong Zhou, Jie Zhang, Lin Zhou, Ning Su, Hanxiao Zhu, Yide Yang

**Affiliations:** 1 School of Physical Education Shenzhen University Shenzhen China; 2 School of Physical Education and Sport Science Fujian Normal University Fuzhou China; 3 School of Physical Education Hebei Normal University Shijiazhuang China; 4 School of Medicine Hunan Normal University Changsha China

**Keywords:** eHealth literacy, measurement, Web 3.0, psychometric properties, NCD, older adults, noncommunicable diseases, Electronic Health Literacy, health literacy, eHealth, reliability, validity

## Abstract

**Background:**

In the current digital era, eHealth literacy plays an indispensable role in health care and self-management among older adults with noncommunicable diseases (NCDs). Measuring eHealth literacy appropriately and accurately ensures the successful implementation and evaluation of pertinent research and interventions. However, existing eHealth literacy measures focus mainly on individuals’ abilities of accessing and comprehending eHealth information (Web1.0), whereas the capabilities for web-based interaction (Web2.0) and using eHealth information (Web3.0) have not been adequately evaluated.

**Objective:**

This study aimed to examine the reliability, validity, and measurement invariance of the eHealth Literacy Scale-Web3.0 (eHLS-Web3.0) among older adults with NCDs.

**Methods:**

A total of 642 Chinese older adults with NCDs (mean age 65.78, SD 3.91 years; 55.8% female) were recruited in the baseline assessment, of whom 134 (mean age 65.63, SD 3.99 years; 58.2% female) completed the 1-month follow-up assessment. Baseline measures included the Chinese version of the 24-item 3D eHLS-Web3.0, the Chinese version of the 8-item unidimensional eHealth Literacy Scale (eHEALS), and demographic information. Follow-up measures included the 24-item eHLS-Web3.0 and accelerometer-measured physical activity and sedentary behavior. A series of statistical analyses, for example, Cronbach α, composite reliability coefficient (CR), confirmatory factor analysis (CFA), and multigroup CFA, were performed to examine the internal consistency and test-retest reliabilities, as well as the construct, concurrent, convergent, discriminant, and predictive validities, and the measurement invariance of the eHLS-Web3.0 across gender, education level, and residence.

**Results:**

Cronbach α and CR were within acceptable ranges of 0.89-0.94 and 0.90-0.97, respectively, indicating adequate internal consistency of the eHLS-Web3.0 and its subscales. The eHLS-Web3.0 also demonstrated cross-time stability, with baseline and follow-up measures showing a significant intraclass correlation of 0.81-0.91. The construct validity of the 3D structure model of the eHLS-Web3.0 was supported by confirmatory factor analyses. The eHLS-Web3.0 exhibited convergent validity with an average variance extracted value of 0.58 and a CR value of 0.97. Discriminant validity was supported by CFA results for a proposed 4-factor model integrating the 3 eHLS-Web3.0 subscales and eHEALS. The predictive validity of the eHLS-Web3.0 for health behaviors was supported by significant associations of the eHLS-Web3.0 with light physical activity (β=.36, *P*=.004), moderate to vigorous physical activity (*β*=.49, *P*<.001), and sedentary behavior (*β*=–.26, *P*=.002). Finally, the measurement invariance of the eHLS-Web3.0 across gender, education level, and residence was supported by the establishment of configural, metric, strong, and strict invariances.

**Conclusions:**

The present study provides timely empirical evidence on the reliability, validity, and measurement invariance of the eHLS-Web3.0, suggesting that the 24-item 3D eHLS-Web3.0 is an appropriate and valid tool for measuring eHealth literacy among older adults with NCDs within the Web3.0 sphere.

## Introduction

### Background

Noncommunicable diseases (NCDs), known as chronic diseases, result in the mortality of 41 million people annually, equivalent to approximately 74% of all global deaths [1]. Characterized by high morbidity, high mortality, low control rates, and limited awareness, NCDs impose a considerable financial burden on individuals, their families, and society as a whole, particularly among older patients [2]. In China, the prevalence rate of NCDs among older adults aged 60 years and older was 50%-75%, as reported in recent epidemiological studies [3-5]. Therefore, NCDs in older adults are a vital public health concern, and their management is a global challenge.

Previous evidence has demonstrated that empowering and educating patients with NCDs to focus on self-management and health promotion is essential [2,6]. Enabling patients to inquire about their medical status, comply with medication instructions, enhance their engagement and compliance in the health care process, adopt healthier lifestyles, and ultimately reduce reliance on constant supervision from health care professionals is a challenging task [6,7]. Nevertheless, facilitating patient self-care is a critical step toward improving the overall health status and alleviating the burden on health care facilities, especially within low- and middle-income countries [7,8].

With the rapid advancement of technology, the internet has become the quickest and most easily accessible resource for obtaining and delivering health information, offering ample opportunities for self-management and health promotion [6,9]. Recent review studies have consistently shown that internet-based health interventions for individuals with NCDs can have a substantial impact on enhancing self-management and patient engagement and compliance with their health care [10,11]. Despite the potential of the internet to improve health care services for NCDs, older adults encounter significant challenges in using digital health technologies [12]. In particular, the information found on the internet originates from numerous providers and sources that are difficult to regulate, thereby leading to potential problems in terms of accuracy and the potential dissemination of prejudiced content that aligns with the interests and objectives of certain parties involved [13]. Previous research has highlighted the considerable difficulties faced by older adults in accessing reliable and high-quality health information that addresses their specific health needs [14,15]. Furthermore, studies have revealed that a noteworthy proportion of older internet users lack confidence in their capability to execute basic tasks on the internet [15]. The challenges mentioned above not only impede older adults from harnessing the internet’s full potential for health care purposes but also exacerbate the digital divide and health disparities [12]. In such a scenario, eHealth literacy is emphasized in numerous studies as a critical skill that older adults with NCDs must acquire in the digital era of disease management and health care [2,6,16].

eHealth literacy, first proposed by Norman and Skinner in 2006 [17], refers to “individual’s abilities to seek, find, understand, and appraise health information from electronic resources and apply that knowledge to solve a health problem or make a health-related decision.” The concept of eHealth literacy is founded on social cognitive theory, consisting of 6 essential skills or literacies: traditional literacy, health literacy, information literacy, scientific literacy, media literacy, and computer literacy [17,18]. To provide a general assessment of this concept that can assist in clinical decision-making and health promotion planning for individuals or specific samples, Norman and Skinner [17] developed an 8-item unidimensional eHealth Literacy Scale (eHEALS). The eHEALS is the most well-known and extensively used instrument for assessing eHealth literacy to date [19]. The reliability and validity of the eHEALS have been extensively examined in diverse cultural contexts, including English [17,20], German [21], Spanish [22], Dutch [23], Italian [24], Portuguese [25], Japanese [26], and Chinese [27], providing compelling evidence of its efficacy across multiple languages and cultures.

However, as technology continues to advance, there has been an increasing acknowledgment of the necessity to update the content of eHealth literacy to ensure optimal synchronization with the evolving internet landscape [23,28,29]. Several studies have raised concerns regarding the unidimensional nature of the eHEALS, as well as its inadequate performance in psychometric evaluations, particularly when using it to measure the usage of novel technologies in seeking and assessing health information [23,27-30]. For example, previous research has shown a weak association between eHEALS and eHealth behaviors beyond web-based information-researching skills, indicating the requirement to update the tool [23]. Furthermore, a recent systematic review indicated that the structure of the eHEALS varied across multiple studies, where a 2-factor or 3-factor structure was also identified in certain studies [19]. There has also been some questioning of the variability of the items, even though the eHEALS had the same factor construct [19]. Alongside the influence of cultural contexts, a primary reason for the inconsistencies of the factor structures and corresponding items may be that the eHEALS is outdated for use in evaluating eHealth literacy in the new digital age [19].

Indeed, the arguments outlined above are reasonable. As per the widely acknowledged generation divisions of internet evolution, the present internet landscape has progressed through 3 distinct phases, starting with Web1.0 (a read-only web) to Web2.0 (ie, a read-write mode that provides a participatory social web with increased collaboration and interaction among consumers, programmers, service providers, and organization) and to current Web3.0 (ie, a read-write-execute mode that provides digital, personalized, and intelligent services; also known as semantic web) [23,30,31]. The eHEALS was developed 15 years ago for measuring individuals’ capability related to reading and viewing within a Web1.0 context, and therefore, it is necessary to update it to effectively scale current eHealth usage.

To fill this gap, numerous new measurement tools of eHealth literacy have been developed. One example is the 20-item extended version of the eHEALS (eHEALS-E) created by Petrič et al [32], which is designed to better encompass the complicated factors contributing to eHealth literacy. However, the eHEALS-E is based on the same definition as the original eHEALS, and therefore, it may also have limitations in measuring only a narrow aspect of eHealth literacy [19]. Furthermore, second-generation instruments of eHealth literacy have been developed (eg, eHealth Literacy Scale [eHLS], Digital Health Literacy Instrument [DHLI], Transactional eHealth Literacy Instrument, eHealth Literacy Assessment Toolkit, and Chinese version of the electronic eHealth Literacy Scale [C-eHEALS]) to assess a broader spectrum of eHealth literacy concepts, ensuring their relevance in the age of social media and eHealth [19,28]. These measures have provided novel approaches for evaluating eHealth literacy, with some of them specifically designed to measure web communication capabilities. However, their coverage is limited to Web2.0 skills, and evaluation of eHealth literacy content relevant to Web3.0 technologies remains absent [33]. Recently, Liu et al [33] developed a 24-item eHealth Literacy Scale-Web3.0 (eHLS-Web3.0) to measure eHealth skills in the context of Web3.0. Compared with previous eHEALS and second-generation eHealth literacy scales, the eHLS-Web3.0 is an improvement consisting of 3 distinct dimensions (ie, acquisition, verification, and application) that evaluates the abilities covering the entire spectrum of Web1.0 (eg, searching, understanding, and identifying), 2.0 (eg, sharing and interactive communication), and 3.0 (eg, recording, self-managing, applying, and adjusting) [33]. The reliabilities, validities, and measurement invariance of the eHLS-Web3.0 across gender and region have been supported in a previous study with young adults [33], whereas its psychometric properties remain unexplored in older adults, especially those living with NCDs. Therefore, further research is needed to investigate the applicability and psychometric properties of the eHLS-Web3.0 in older adults with NCDs, which may inform the development of effective interventions to improve eHealth literacy and promote better health care outcomes in this population.

### This Study

Given the above, the purpose of this study was to examine the reliability, validity, and measure invariance of the eHLS-Web3.0 in a sample of older adults with NCDs. In particular, this study has 3 main objectives. First, the internal consistency and test-retest reliabilities of the eHLS-Web3.0 would be examined. Based on previous studies [12,17,20,34-36], a conventional 1-month time frame for evaluating the test-retest reliability was used in this study. Second, the construct, convergent, concurrent, discriminant, and predictive validities of the 24-item 3D eHLS-Web3.0 would be ascertained. Because the eHEALS has been proven to be a reliable tool for measuring eHealth literacy among older adults with NCDs in previous studies [2,17], this study would use the eHEALS as the criteria scale for the examination of the concurrent validity of the eHLS-Web3.0. Furthermore, considering the content distinction between the eHLS-Web3.0 and the eHEALS, the discriminant validity of the eHLS-Web3.0 would be examined by comparing the eHLS-Web3.0 subscales and the eHEALS. Additionally, previous studies have established a strong association between eHealth literacy and various health behaviors [13,37]. Specifically, eHealth literacy has been shown to positively correlate with health-promoting behaviors (eg, physical activity) and negatively correlate with risk behaviors (eg, sedentary behavior) among young and older adults [38,39]. Therefore, this study would investigate the predictive validity of the eHLS-Web3.0 for 2 specific health behaviors (ie, physical activity and sedentary behavior), given their crucial impact on the physical and mental well-being of older adults with NCDs [40]. By identifying the predictive validity of the eHLS-Web3.0, this study is expected to make a noteworthy contribution to future research in this field. Finally, considering that gender, education level, and residence are potential correlates of eHealth literacy [6,33,41], the measurement invariance of the eHEALS would be examined at the configural, metric, strong, and strict levels across gender, education level, and residence.

## Methods

### Design, Participants, and Procedure

This study applied a 2-wave prospective design. Considering an item-to-response ratio of 1:10 and the recommendation for a minimum sample size of 200 in confirmatory factor analysis (CFA) [42], 240 participants were required to ensure a robust statistical estimation. With an approximate response rate of 85% and a prior estimated prevalence rate of NCDs of 50% in older adults [4], a minimum of 564 participants were required to be contacted at the initial recruitment stage. Eligible participants for the study were older adults who met the following inclusion criteria: (1) aged 60 years or older, (2) experiencing at least 1 type of NCD (eg, cardiovascular diseases, cancer, type 2 diabetes, and obesity), (3) no physical mobility restrictions, (4) no cognitive disorders, (5) proficient in reading and understanding Chinese, and (6) having access to a smartphone or laptop.

Participants were recruited from the outpatient departments of 6 hospitals from 3 cities (Shiyan, Wuhan, and Suizhou) of Hubei Province (Central China) using a convenience sampling approach. The survey was implemented using the SOJUMP web-based survey platform (Changsha Ranxing Information Technology Co, Ltd). Two health care professionals undertook an initial review of the scale items to ensure that the wording was appropriate for older adults with NCDs. Subsequently, 6 older adults with NCDs (3 female and 3 male) were invited to complete a pilot assessment aimed at (1) optimizing the design of the electronic questionnaires (eg, using the large font and highlighting the key information) and (2) refining the language and eliminating any errors to ensure that the scale items were easily comprehensible for the target population.

In the main study, participants were provided with a QR code through nurses to gain access to the web-based survey. Before answering the questionnaires, participants were required to sign an informed consent form on the first page of the survey. The web-based survey lasted approximately 20 minutes. To ensure a robust evaluation for the scale test-retest reliability and predictive validity, a minimum of 100 participants were needed [43]. Accounting for a potential 30% attrition rate (eg, no response and invalid or missing data) [4], a total of 142 participants were required for the second-wave data collection. Invitations were sent out randomly via SMS text messages to those who had completed the first round of data collection until enough participants agreed to participate in the follow-up survey, scheduled for 1 month later. Participants who agreed to participate in the second round of investigation were requested to revisit the hospital, where 2 qualified assistants guided them to complete the follow-up web-based survey and provided detailed instructions on the use of the accelerometer for data collection.

### Ethical Considerations

This study adhered to the principles outlined in the Declaration of Helsinki by the World Medical Association. The Medical Ethics Committee of the Faculty of Medicine at Shenzhen University reviewed and approved this study (PN-202300066). All participants provided signed informed consent for both the primary study and the sensitivity analyses. The data were anonymized to protect participant privacy, and participation in the study was entirely voluntary. As a token of appreciation, participants received a participation fee of 5 RMB (US $0.7) on completing the data collection.

### Measures

#### eHealth Literacy Scale-Web3.0

The 24-item eHLS-Web3.0 was originally developed by Liu et al [33] for the Chinese adult population. This scale comprises 3 dimensions: acquisition (items 1-4 and 11-14), verification (items 5-10), and application (items 15-24). Responses were indicated on a 5-point Likert scale, ranging from 1 (strongly disagree) to 5 (strongly agree). The total score of the scale ranges from 24 to 120, with a higher score indicating a greater level of eHealth literacy. The reliability and validity of the eHLS-Web3.0 have been fully supported by previous research with Chinese young adults (Cronbach α=0.91-0.97).

#### eHEALS

The 8-item eHEALS was developed by Norman and Skinner [17] for use among Canadian adolescents. The original scale is unidimensional and has been validated in various countries across diverse populations. The Chinese version of the 8-item eHEALS has been examined in previous studies among older adults with NCDs, where the reliability and validity of the scale have been fully supported (Cronbach α=0.95-0.98).

#### Health Behaviors

Physical activity and sedentary behavior were measured using the ActiGraph GT3X+ (ActiGraph) on the right side of the waist for 7 consecutive days, with the exception of swimming, bathing, and sleeping time. The accelerometer sampling interval was set at 60-second epochs with a sampling frequency of 30 Hz. Nonwear time was defined by an interval of 60 consecutive minutes of 0 counts per minute, allowing for 2 minutes of nonzero count interruptions. Participants with at least 3 valid days of accelerometer use (2 weekdays and 1 weekend day) and a minimum wear time of 10 hours per day were eligible for inclusion in the data analysis. The Freedson cutoff point was used for categorizing light physical activity (100-1951 counts/minute), moderate to vigorous physical activity (>1951 counts/minute), and sedentary behavior (<100 counts/minute) [44].

#### Demographics

The demographic information included age, sex, marital status, education level, residence, monthly income, living situation, and BMI.

### Statistical Analyses

The data analyses were performed using IBM SPSS Statistics (version 28.0; IBM Corp) and Mplus 8 (Muthén & Muthén). Data screening and diagnosis tests of data distribution (eg, mean, SD, skewness, and kurtosis) and missing patterns were performed before the descriptive analysis and scale validation. To ensure a reliable estimation for the multidimensional scale, both Cronbach α and composite reliability (CR) coefficients were calculated to evaluate the internal consistency reliability of the eHLS-Web3.0. Additionally, the test-retest reliability was estimated using the intraclass correlation coefficient of pre- and 1-month follow-up data.

The construct validity of the eHLS-Web3.0 was evaluated using CFAs with maximum likelihood estimation. Several goodness-of-fit indices were computed, including robust chi-square (*χ*^2^_R_), robust chi-square to degrees of freedom ratio (*χ*^2^_R_/*df*), comparative-fit index (CFI), Tucker-Lewis index (TLI), root-mean-square error of approximation (RMSEA) and its 90% CI, and standardized root-mean-square residual (SRMR). The following criteria were considered for a satisfactory model goodness of fit: ≤3 for *χ*^2^_R_/*df*, ≥0.9 for CFI and TLI, and ≤0.08 for RMSEA and SRMR [45].

Convergent validity was assessed by examining the average variance extracted (AVE) and CR for each subscale, with AVE >0.5 and CR >0.7 indicating satisfactory convergent validity for the scale. Concurrent validity was assessed by calculating the zero-order correlations of the eHLS-Web3.0 and its subscales with the eHEALS, adjusted for all demographic confounders. For the discriminant validity, a presumptive 4-factor model incorporating 3 eHLS-Web3.0 subscales and unidimensional eHEALS was estimated in the CFA. The discriminant validity of the subscales was confirmed if the 95% CI of the association between these subscales did encompass the value of 0 and if the Wald chi-square test demonstrated a significant change in model fit after removing a constraint that fixed the factor correlation to zero [45]. Additionally, structural equation models were performed to assess the predictive validity of the scale by estimating its association with health behaviors, including physical activity and sedentary behavior.

With a sequential model testing approach, multigroup CFA was used to examine the measurement invariance of the eHLS-Web3.0 across gender, education, and residence. Four distinctive levels of measurement invariance were examined by progressively constraining the parameter estimates of the models to be equivalent across the samples: (1) configural invariance, where no parameter estimates were restricted to equality; (2) metric invariance, where factor loadings were constrained to equality; (3) strong invariance, where both factor loadings and item intercepts were constrained to equality; and (4) structural and strict invariance, where all factor loadings, item intercepts, and factor variance and covariance were restricted to equality. The measure invariance was supported if the change in the value of CFI and RMSEA was ≤0.01 and ≤0.015, respectively [45,46].

## Results

### Descriptive Information of the Study Sample

**Figure 1:**
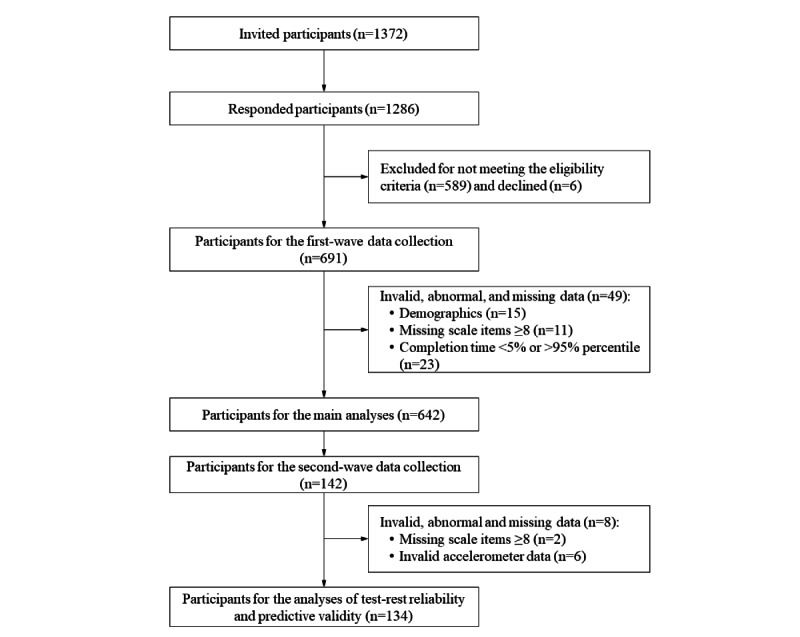
Study process diagram.

As outlined in Figure 1, a total of 642 eligible participants (mean 65.78, SD 3.91 years; 55.8% female) were included in the data analysis, of whom 134 (mean 65.63, SD 3.99 years; 58.2% female) provided valid data at the follow-up assessment. From the diagnostic evaluation, there were no missing data for eHLS-Web3.0 and eHEALS items in the study sample. All the scale items adhered to the normality distribution with absolute values of skewness and kurtosis <1. Descriptive information of the study sample is shown in Table 1.

**Table 1:** Descriptive characteristics of the study sample.

Demographic information			Main study (n=642)		Test-retest (n=134)
Age (years), mean (SD)			65.78 (3.91)		65.63 (3.99)
Age range (years)			60-74		60-74
**Gender, n (%)**					
	Female	358 (55.8)		73 (54.5)	
	Male	284 (44.2)		61 (45.5)	
**Marital status, n (%)**					
	Married	597 (93)		123 (91.8)	
	Single, divorced, or widowed	45 (7)		11 (8.2)	
**Education level, n (%)**					
	Primary	121 (18.8)		26 (19.4)	
	Middle and high school	134 (20.9)		21 (15.7)	
	College and above	387 (60.3)		87 (64.9)	
**Residence, n (%)**					
	Urban	394 (61.4)		82 (61.2)	
	Rural	248 (38.6)		52 (38.8)	
**Monthly income (RMB), n (%)**					
	≤1000 (US $138.1)	68 (10.6)		15 (11.2)	
	1001-3000 (US $138.2-$414.2)	149 (23.2)		29 (21.6)	
	3001-5000 (US $414.3-$690.3)	166 (25.9)		37 (27.6)	
	5001-7000 (US $690.4-$966.4)	186 (29.0)		35 (26.1)	
	≥7001 (US $966.5)	73 (11.4)		18 (13.4)	
**Living situation, n (%)**					
	Living with others	575 (89.6)		117 (87.3)	
	Living alone	67 (10.4)		17 (12.7)	
**Number of NCDs^a^**					
	1	531 (82.7)		108 (80.6)	
	2-3	93 (14.5)		17 (12.7)	
	>3	18 (2.8)		9 (6.7)	
BMI (kg/m^2^), mean (SD)			22.95 (2.82)		22.46 (2.41)
**BMI category, n (%)**					
	Underweight	36 (5.6)		6 (4.5)	
	Normal weight	410 (63.9)		87 (64.9)	
	Overweight and obese	196 (30.5)		41 (30.6)	
**Health behavior, mean (SD)**					
	LPA^b^ (minutes/day), range=45-420	N/A^c^		145.93 (89.42)	
	MVPA^d^ (minutes/day), range=4-98	N/A		26.82 (24.40)	
	Sedentary behavior (minutes/day), range=175-865	N/A		573.49 (135.46)	

^a^NCD: noncommunicable disease.

^b^LPA: light physical activity.

^c^N/A: not applicable.

^d^MVPA: moderate to vigorous physical activity.

### Reliabilities of the eHLS-Web3.0 in the Study Sample

Table 2 shows the mean value, SD, score range, and internal consistency and test-retest reliabilities of the eHLS-Web3.0 in the study sample. Regarding the internal consistency reliability, the eHLS-Web3.0 and its 3 subscales exhibited adequate Cronbach α values (range=0.89-0.94) and CR coefficients (range=0.90-0.97). Regarding the test-retest reliability, 2 time-point measures showed a strong intraclass correlation for the eHLS-Web3.0 and 3 subscales of the eHLS-Web3.0 (*r*=0.81-0.91).

**Table 2:** Mean (SD), score range, Cronbach α, composite (ρ), and test-retest (*r*) reliabilities of the 24-item eHealth Literacy Scale-Web3.0 (eHLS-Web3.0) in the study sample (n=642).

Scale			Mean (SD)		Range		α		ρ		*r* (n=134)
**eHLS-Web3.0**			78.61 (11.1)		12-40		0.94		0.97		0.91
	Acquisition	27.14 (4.26)		9-30		0.92		0.91		0.87	
	Verification	19.89 (3.67)		20-50		0.89		0.90		0.81	
	Application	31.57 (5.08)		42-115		0.93		0.93		0.90	

### Validities of the eHLS-Web3.0 in the Study Sample

For the constructive validity, the results of the CFA showed that the 24-item 3D eHLS-Web3.0 achieved the criteria for good model fit indices in the study sample, with *χ*^2^_R_=674.4, *df*=248, *χ*^2^_R_/*df*=2.72 (<3), CFI=0.952 (>0.9), TLI=0.946 (>0.9), RMSEA=0.052 (90% CI 0.047-0.056; <0.08), and SRMR=0.034 (<0.08). The standardized factor loadings of the eHLS-Web3.0 items ranged from 0.658 to 0.819 (see Table S1 in Multimedia Appendix 1).

For concurrent validity, the zero-order correlations between the eHLS-Web3.0 subscales and eHEALS were significant (*r=*0.47-0.76), indicating a satisfactory result, as outlined in Table 3. The AVE and CR were calculated as 0.58 and 0.97, respectively, using the standardized factor loadings of the items, showing a satisfactory convergent validity of the eHLS-Web3.0.

The latent factor correlations in a proposed 4-factor CFA model (ie, 3 eHLS-Web3.0 subscales and eHEALS) were calculated to evaluate the discriminant validity of the eHLS-Web3.0 subscales and eHEALS in the study sample. The goodness-of-fit indices of the 4-factor model were inferior to those of the original 3-factor eHLS-Web3.0 model, with *χ*^2^_R_=2079.8, *df*=461, *χ*^2^_R_/*df*=4.51, CFI=0.874, TLI=0.865, RMSEA=0.074 (90% CI 0.071-0.077), and SRMR=0.078. Table 4 presents the statistical results of the discriminant validity analysis. Wald chi-square findings were statistically significant in the study sample (all *P*<.001), and the CIs for each correlation did not contain the value of 0, demonstrating a satisfactory discriminant validity of the eHLS-Web3.0.

In addition, the eHLS-Web3.0 significantly predicted light physical activity (*β*=.36, 95% CI 0.19-0.53; *P*=.004), moderate to vigorous physical activity (*β*=.49, 95% CI 0.35-0.62; *P*<.001), and sedentary behavior (*β*=–.26, 95% CI–0.40 to –0.12; *P*=.002), supporting the predictive validity of the scale for health behaviors. The goodness-of-fit indices indicated a satisfactory result for the 3 models, with *χ*^2^_R_=398.3-403.8, *df*=271, *χ*^2^_R_/*df*=1.47-1.49, CFI=0.949-0.951, TLI=0.944-0.946, RMSEA=0.059-0.060, and SRMR=0.043-0.044.

**Table 3:** Zero-order correlation between the subscales of the 24-item eHealth Literacy Scale-Web3.0 (eHLS-Web3.0) and 8-item eHealth Literacy Scale (eHEALS), n=642.

Scale	eHEALS	eHLS-Web3.0	Acquisition	Verification	Application
eHEALS	1				
eHLS-Web3.0	0.69^a^	1			
Acquisition	0.76^a^	0.87^a^	1		
Verification	0.48^a^	0.79^a^	0.56^a^	1	
Application	0.57^a^	0.88^a^	0.64^a^	0.52^a^	1

^a^*P*<.001.

**Table 4:** Latent interfactor correlations and discriminant validity statistics for the 24-item eHealth Literacy Scale-Web3.0 in the study sample (n=642).

Scale	Interfactor correlation	95% CI	Wald chi-square test^a^
Acquisition↔Verification	0.86	0.84-0.88	197.4^b^
Acquisition↔Application	0.91	0.89-0.92	155.8^b^
Verification↔Application	0.84	0.82-0.86	159.0^b^
Acquisition↔eHEALS^c^	0.94	0.93-0.95	62.3^b^
Verification↔eHEALS	0.82	0.79-0.84	151.7^b^
Application↔eHEALS	0.85	0.83-0.87	113.6^b^

^a^Wald chi-square test: Wald chi-square test constraining the values of the latent interfactor correlations to zero.

^b^*P*<.001.

^c^eHEALS: 8-item eHealth Literacy Scale.

### Measurement Invariance of the eHLS-Web3.0 in the Study Sample

Table 5 presents the results of the examination regarding the measurement invariance of the eHLS-Web3.0 across gender, education level, and residence. The configural, metric, strong, and strict models were all shown to have a satisfactory fit to the data for all 3 pairs of subsamples, with ΔCFI <0.01 and ΔRMSEA <0.015. These indices provide support for the invariance of the factorial construct, factor loadings, intercepts, and residual variance of the eHLS-Web3.0 across gender, education level, and residence.

**Table 5:** Measurement invariance of the 24-item eHealth Literacy Scale-Web3.0 across gender, education level, and residence.

Model			Chi-square^a^ (*df*)		CFI^b^		ΔCFI^c^		RMSEA^d^		RMSEA 90% CI		ΔRMSEA^e^
**Female (n=358)—Male (n=254)**													
	M0^f^	989.0 (496)		0.944		N/A^g^		0.056		0.051-0.061		N/A	
	M1^h^	1011.6 (517)		0.944		0		0.055		0.050-0.060		–0.001	
	M2^i^	1068.1 (541)		0.940		–0.004		0.055		0.050-0.060		0	
	M3^j^	1072.2 (547)		0.940		0		0.055		0.050-0.050		0	
**Below college (n=255)—College and above (n=387)**													
	M0	966.9 (496)		0.948		N/A		0.054		0.049-0.059		N/A	
	M1	993.0 (517)		0.947		–0.001		0.054		0.049-0.059		0	
	M2	1017.6 (541)		0.947		0		0.052		0.047-0.057		–0.002	
	M3	1026.8 (547)		0.946		–0.001		0.052		0.047-0.057		0	
**Rural (n=248)—Urban (n=394)**													
	M0	955.3 (496)		0.947		N/A		0.054		0.049-0.059		N/A	
	M1	978.5 (517)		0.947		0		0.053		0.048-0.058		0.001	
	M2	1027.5 (541)		0.944		–0.003		0.053		0.048-0.058		0	
	M3	1056.4 (547)		0.941		–0.003		0.054		0.049-0.059		–0.001	

^a^Chi-square: robust chi-square.

^b^CFI: comparative fit index.

^c^ΔCFI: change in the CFI.

^d^RMSEA: root-mean-square error of approximation.

^e^ΔRMSEA: change in the RMSEA.

^f^M0: baseline configural invariance model.

^g^N/A: not applicable.

^h^M1: metric invariance model.

^i^M2: strong invariance model.

^j^M3: strict invariance model.

## Discussion

### Principal Findings

This study aimed to evaluate the reliability, validity, and measurement invariance of the eHLS-Web3.0 for use with older adults who are living with NCDs. In particular, this study examined the internal consistency and test-retest reliabilities, as well as the construct, concurrent, convergent, discriminant, and predictive validities, and the measurement invariance of the eHLS-Web3.0 across gender, education level, and residence. Overall, the results from this study suggest that the eHLS-Web3.0 is a reliable and valid tool for measuring eHealth literacy in Chinese older adults with NCDs.

Regarding the reliabilities, analyses of the Cronbach α and CR coefficients indicated adequate internal consistency reliability for both the eHLS-Web3.0 and its 3 subscales. These findings are consistent with previous research on the use of the eHLS-Web3.0 among Chinese young adults [33]. It is worth noting that while previous studies have generally supported the reliability of the eHEALS and other eHealth literacy assessments (eg, DHLI and C-eHEALS) among older adults or those with NCDs [2,27,28], the novel eHEALS-Web3.0 tool has not yet been evaluated for reliability in older populations. This study is the first to investigate the reliability of the eHEALS-Web3.0 among older adults with NCDs. Additionally, previous psychometric analyses of eHealth literacy measures have primarily focused on internal consistency reliability, with test-retest reliability often overlooked [19,27,28]. In contrast, this study further examined the test-retest reliability of the eHLS-Web3.0 and its subscales, and the findings demonstrated a strong cross-time stability for the scale, as evidenced by a significant correlation between baseline and 1-month follow-up measures.

Regarding the construct validity, the CFA results provided support for the 3D model structure of the eHLS-Web3.0 among Chinese older adults with NCDs. The acquisition and verification subscales of the eHEALS-Web3.0 assess individuals’ eHealth abilities in Web1.0 and Web2.0 contexts, similar to the eHEALS and second-general eHealth literacy measures [12,19,27]. However, the eHEALS-Web3.0 stands out by also evaluating individuals’ proficiency in applying eHealth information to evolving health needs in the Web3.0 era (ie, the application subscale). As the digital landscape advances, individuals have more opportunities and options to use eHealth information. For instance, they can use eHealth information to make informed health decisions or resolve health-related problems, create their own health data, monitor their health status, interact with others, exchange information, and provide health advice to other health information seekers [31,33]. The 3D eHLS-Web3.0 provides a comprehensive assessment of eHealth literacy, catering to the present digital circumstances.

For other validities of the eHLS-Web3.0, the concurrent validity was confirmed by a significant correlation between the eHLS-Web3.0 and its subscales with the eHEALS, while the AVE and CR supported the convergent validity of the scale. In addition, as the 3 eHLS-Web3.0 subscales and eHEALS differ in conceptual content, a 4-factor model integrating the acquisition, verification, and application subscales and unidimensional eHEALS was established to confirm the discriminant validity of the scale. Although the Wald chi-square test results supported the discriminant validity of the eHLS-Web3.0, a high correlation was observed among the latent factors in the 4-factor model, possibly due to measurement errors [47]. To validate the earlier findings, zero-order correlations were calculated using composite (averaged) scales. Fortunately, the overall results confirmed the discriminant validity of the scale. Finally, the predictive validity of the eHLS-Web3.0 was supported by a significant positive association between the eHLS-Web3.0 and physical activity, as well as a negative association with sedentary behavior. Previous studies have demonstrated a positive association between eHealth literacy and health-promoting behaviors (eg, physical activity) among diverse populations [13,37,48]. However, there is a lack of evidence on the relationship between eHealth literacy and risk behaviors (eg, sedentary behavior). Our findings underline the potential of including eHealth literacy as a modifiable factor in future eHealth interventions to facilitate health behaviors and improve health outcomes among older adults with NCDs.

For measurement invariance, the establishment of configural, metric, strong, and strict invariances demonstrated that the eHLS-Web3.0 is a psychometrically sound instrument for measuring eHealth literacy among Chinese older adults with NCDs, regardless of their gender, education level, and residence. These invariances provide a solid foundation for making appropriate and meaningful transgroup comparisons in future studies.

### Limitations and Implications

Some limitations should be noted. First, the nonrandom sampling used in this study may have limited the representativeness of the study findings. Therefore, a stratified random sampling approach is warranted in future studies. Second, given that the study findings are based only on the sample of Chinese older adults with NCDs, one should be cautious when generalizing these results to other samples. Future studies should examine the psychometric properties of the scale across different populations and diverse cultural contexts. Moreover, self-reported measures may result in some response biases (eg, recall bias and social desirability); therefore, the inclusion of objective means for assessing eHealth literacy should be considered in the future. Additionally, it is worth exploring the prediction of eHealth literacy on other health outcomes and examining its underlying mechanisms. Finally, from a pragmatic perspective, it may be beneficial to develop and validate a brief version of the eHLS-Web3.0, particularly with regard to older populations who may struggle with completing lengthy self-reported scales.

Despite the aforementioned limitations, this study addresses a significant gap in the literature by validating and applying the eHLS-Web3.0, a specific measure of eHealth literacy used for Chinese older adults with NCDs in the Web3.0 landscape. Previous reviews have revealed a wide range of influential factors of eHealth literacy as well as a positive correlation between higher eHealth literacy and better health behaviors, knowledge, and attitudes in older adults [38,49-51]. These findings indicate the potential for developing eHealth literacy interventions to promote positive health behaviors in the future while considering various socioeconomic and cultural variables. However, previous studies have yielded conflicting results regarding certain physical and psychosocial outcomes [38], underlining the need for more high-quality research. It is important to note that the success of these efforts largely depends on a reliable and accurate assessment of eHealth literacy [52].

The findings of this study provide robust support for the reliability, validity, and measurement invariance of the eHLS-Web3.0, indicating that this up-to-date tool can be widely used in future research endeavors to appropriately and accurately assess older adults’ abilities to search for, retrieve, evaluate, and use web-based health resources. This advancement has the potential to significantly contribute to both the field of eHealth literacy research and the development of targeted health promotion programs in the future. As digital technology increasingly infiltrates the health care sector, promoting eHealth literacy among older adults is more critical than ever [52,53]. The development and validation of the eHLS-Web3.0 marks a significant milestone in the field of eHealth literacy research, serving as a necessary foundation for future empirical investigations and targeted interventions aimed at improving eHealth literacy among older adults.

### Conclusions

To the best of our knowledge, this is the first study to examine the psychometric properties and measurement invariance of the eHLS-Web3.0 among Chinese older adults with NCDs. This study provides evidence for internal consistency and test-retest reliabilities, construct, concurrent, convergent, discriminant, and predictive validities, and the measurement invariance of the 24-item 3D eHLS-Web3.0 for use with Chinese older adults with NCDs. The eHLS-Web3.0 can serve as a psychometrically sound instrument for assessing eHealth literacy in the Chinese context.

## Appendix

Multimedia Appendix 1 Standardized factor loadings of the 24-item eHealth Literacy Scale-Web3.0.

## Abbreviations

AVE: average variance extracted
C-eHEALS: Chinese version of the electronic eHealth Literacy Scale
CFA: confirmatory factor analysis
CFI: comparative fit index
CR: composite reliability
DHLI: Digital Health Literacy Instrument
eHEALS: eHealth Literacy Scale
eHEALS-E: 20-item extended version of the eHEALS
eHLS-Web3.0: eHealth Literacy Scale-Web3.0
NCD: noncommunicable disease
RMSEA: root-mean-square error of approximation
SRMR: standardized root-mean-square residual
TLI: Tucker-Lewis index
