# Supplementary material for: Electronic Health Literacy Scale-Web3.0 for Older Adults with Noncommunicable Diseases: Validation Study
Source: J Med Internet Res. 2024 Jun 3;26:e52457. doi: 10.2196/52457 (PMC11184271; doi:10.2196/52457)
Supplement: Multimedia Appendix 1 [file jmir_v26i1e52457_app1.pdf]

Table S1. Standardized factor loadings of the 24-item eHealth Literacy Scale-Web3.0 in the study sample (n = 642).

	**Acquisition**	**Verification**	**Application**
eHealth Literacy Scale-Web3.0	0.885	0.712	0.802
1. I know where I can find useful health resources on the Internet.<br>我知道网上哪里可以找到有帮助的健康资源。	0.718		
2. I know which kind of eHealth tool I should choose to fit my health needs (i.e., checking drug description, seeking health advice or making health promotion plan).<br>我知道应该选择何种网络健康工具以满足我的健康需求（如：查阅药物说明、进行健康咨询、制定健康促进计划等不同方面的健康需求）	0.706		
3. I can judge whether the eHealth tool is credible or not.<br>我能够判断网络健康工具是否可信。	0.740		
4. I can obtain the health information (e.., information about medical, sport, or daily care) via internet.<br>我能通过网络获取健康信息（如：医疗和运动保健信息等）	0.776		
5. I know how to verify the eHealth information from multiple sources.<br>我知道如何从不同渠道去验证网络健康信息。		0.813	
6. When searching the health information online, I will check the credentials and affiliations of authors.<br>使用网络查询健康信息时，我会去查看作者的资历证书与所属机构。		0.784	
7. When searching the health information online, I will check who owns the website.<br>使用网络查询健康信息时，我会去查看网页的拥有者。		0.745	
8. When searching the health information online, I will check the date of its last update.<br>使用网络查询健康信息时，我会去查看该网络信息最近一次的更新日期。		0.757	
9. When searching the health information online, I will check whether other print or web resources had confirmed this information.<br>使用网络查询健康信息时，我会去查看是否有其他出版物或网络资源确认过此信息。		0.754	
10. Even if the health information I obtained is from someone I trust, I will still verify it on the internet.		0.782	

即使是信任的人告诉我的健康信息，我也会上网去验证它。		
11. I can judge whether the health information online has a commercial interest (e.g., the person providing the information is for the sale of a product).<br>我能够判断网上的信息是否带有商业利益（如：提供该信息的人是为了销售某一产品）。	0.734	
12. When communicating with others online, I can articulate my health-related concerns clearly.<br>在网上与他人交流时，我能够十分清晰的表达自己健康相关的想法。	0.819	
13. When replying to others' health-related help-seeking online, I can provide responsible responses (which means my answer can neither mislead others, nor compromise my own information security).<br>假如我需要在网上回答其他人的健康求助时，我能够给出负责人的回答（即，我的回答不会误导他人，也不会伤害自己的信息安全）。	0.757	
14. When using the eHealth tools, I will protect the originality of the information (e.g., never plagiarize others' original content, report an offence to those infringing others).<br>使用网络健康工具时，我会去维护信息的原创新（如：不盗用他人发布的原创内容，对剽窃行为进行举报等）。	0.777	
15. I know how to use the eHealth tools to record my health behaviors (e.g., physical activity, sleep).<br>我知道如果使用网络健康工具来记录我的健康行为（如：运动和睡眠情况）。		0.776
16. I know how to use the eHealth tools to track my health behaviors (i.e., acquainting my exercise frequency or the change curve of body fat percent).<br>我知道如何利用网络健康工具对我的健康行为进行追踪（如：从以往的使用记录中了解自身运动频率、体脂率等身体指标的变化曲线）		0.752
17. I know how to make use of the records on the eHealth tools to provide reference for my daily health management.<br>我能够利用网络健康工具上的记录信息为我的日常健康管理提供参考。		0.781
18. I can continuously use a certain eHealth tool (e.g., APP, intelligent body fat scale, fitness bracelet) for a long time, if		0.741

needed.<br>若有需要，我能够坚持使用某一网络健康工具（如 APP，体脂秤或手环等）。	
19. I can sue the eHealth tools with a clear plan if needed.<br>若有需要，我能够有计划地使用网络健康工具。	0.804
20. I can adjust my frequency, strength and usage pattern timely when using the eHealth tool according to the actual condition.<br>使用网络健康工具的过程中，我能够根据实际情况调整自己的使用频率、强度与方式。	0.745
21. I know how to use the eHealth tools to post and share my eHealth behaviors (e.g., post my motion trails on health Apps or Moments on WeChat).<br>我知道如何使用社交软件上的运动功能（如微信运动）与他人进行互动（如点赞、助力等）。	0.773
22. I know how to use the sports functions on social network services (e.g., WeRun on WeChat) to interact with others (e.g., thumb up, forward, etc.)<br>我知道如何在网络健康工具上发布和分享自己的健康行为（如：将运动轨迹分享到健康 APP 或朋友圈）。	0.658
23. I will target the advanced players I follow on the eHealth tools, learn from them and catch up with them.<br>我会将我在网络健康工具上所关注的人作为目标，去效仿和赶超他们。	0.750
24. I will try out some health-related suggestions online and control the risks (e.g., get injured or mistake medicine).<br>我会去尝试网上与健康有关的提议，并控制其中的风险（如:受伤或错服药物）。	0.771
